# Supplementary figures and images for: Micronucleus is not a potent inducer of the cGAS/STING pathway
Source: Life Sci Alliance. 2024 Feb 2;7(4):e202302424. doi: 10.26508/lsa.202302424 (PMC10837050; doi:10.26508/lsa.202302424)

Fig S3A

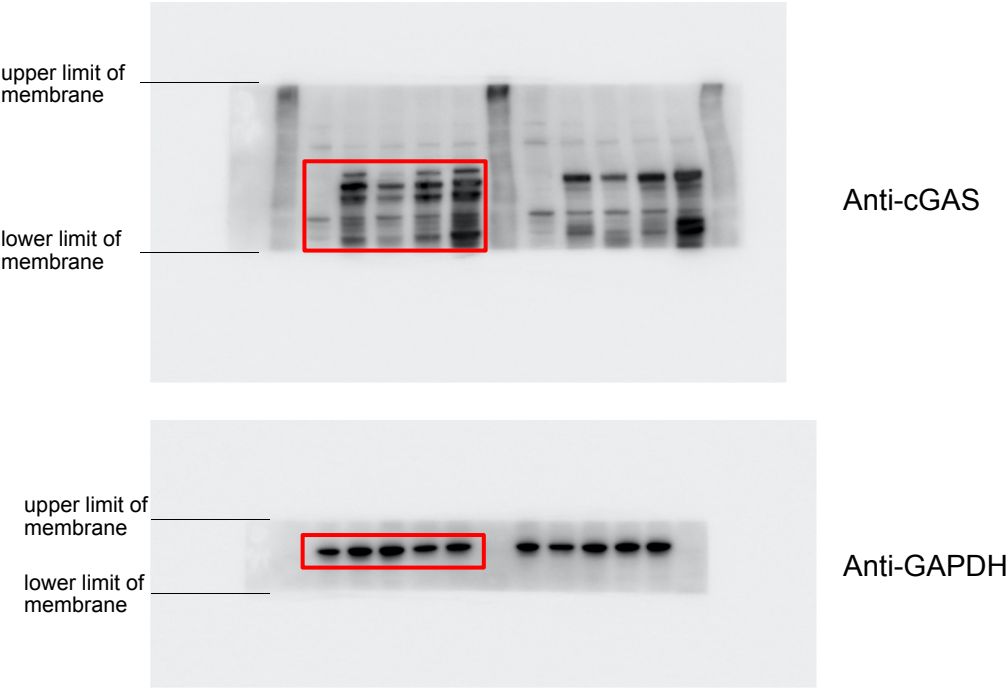

Fig S3D

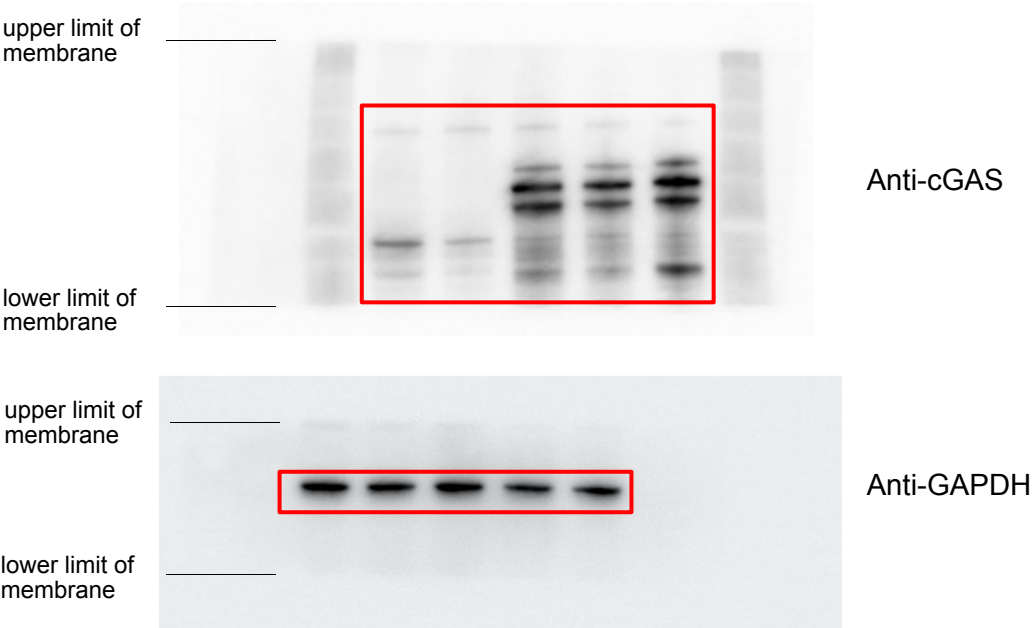

Supplement: Supplementary file 5 [file LSA-2023-02424_SdataFS3.1.pdf]

Fig S6D

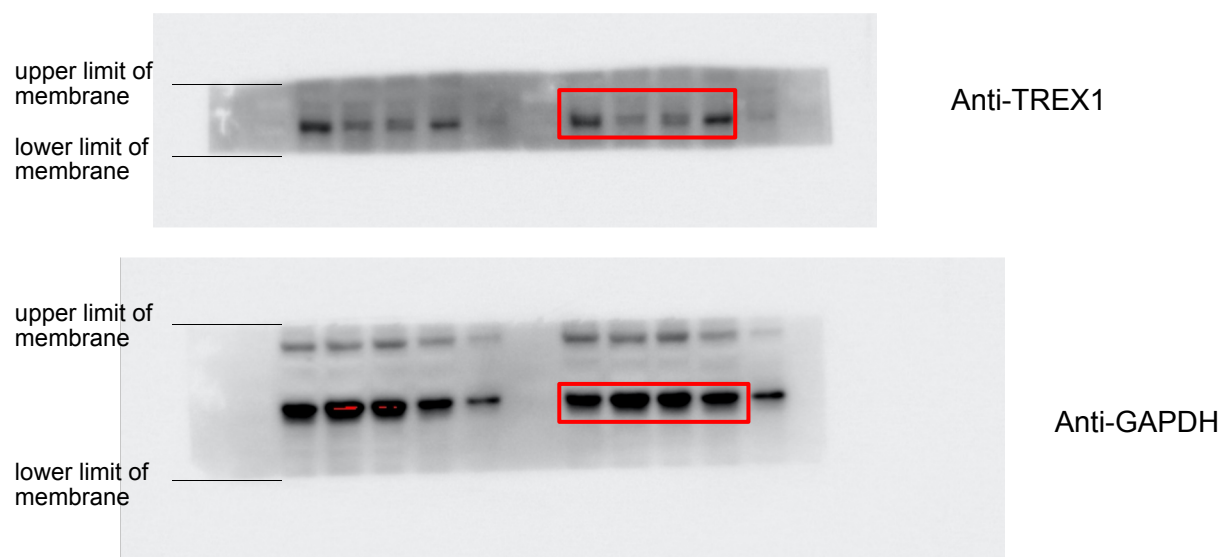

Supplement: Supplementary file 12 [file LSA-2023-02424_SdataFS6.2.pdf]
